# Paratesticular Solitary Fibrous Tumor: A Case Report With Sonographic and Histopathologic Insights

**DOI:** 10.7759/cureus.109443

**Published:** 2026-05-22

**Authors:** Miriam Marques, Bernardo Martins, Tiago Romão, Ana Isabel Belo

**Affiliations:** 1 Radiology, Hospital Beatriz Ângelo, Loures, PRT; 2 Urology, Hospital Beatriz Ângelo, Loures, PRT; 3 Pathology, Hospital Beatriz Ângelo, Loures, PRT

**Keywords:** extrapleural occurrence, left hemiscrotum, mesenchymal neoplasm, paratesticular solitary fibrous tumor, radical orchiectomy, spindle cell neoplasm, surgical case report

## Abstract

Solitary fibrous tumor (SFT) is a rare mesenchymal neoplasm of fibroblastic differentiation that is generally considered benign. It most commonly arises from the pleura, typically presenting as a thoracic mass. Extrapleural occurrence, particularly within the urogenital tract, such as the paratesticular region, is exceedingly rare. The histopathological characteristics of paratesticular SFTs are similar to those observed in tumors arising at other anatomical sites. We report the case of a 26-year-old man presenting with a painless, firm, and mobile paratesticular mass that had progressively enlarged over two years. The lesion was completely excised surgically without perioperative complications. Gross and histopathological examination demonstrated a well-circumscribed lesion composed of uniform spindle cells embedded in a dense collagenous stroma. Immunohistochemical analysis showed diffuse positivity for cluster of differentiation 34 (CD34). These findings supported the diagnosis of paratesticular SFT.

## Introduction

Solitary fibrous tumor (SFT) is a rare mesenchymal neoplasm characterized by fibroblastic differentiation and is generally considered benign. However, a small proportion of cases exhibit malignant behavior, with studies suggesting that approximately 10-15% may undergo malignant transformation [1]. SFTs most commonly occur in middle-aged adults and show no clear sex predilection. Their exact etiology remains uncertain [2].

Classically, SFTs arise from the pleura and are typically identified as thoracic masses. Although extrapleural manifestations are less common, these tumors have been reported in a wide variety of anatomical locations [2]. Genitourinary involvement is particularly rare, with the kidney and bladder being the most commonly affected sites [3]. Paratesticular localization represents an exceptionally uncommon presentation, with only a limited number of cases described in the literature [4].

Despite their unusual location, paratesticular SFTs share histopathological and immunohistochemical characteristics with SFTs arising in more common anatomical sites [2]. Histologically, SFTs are composed of spindle-shaped cells and commonly demonstrate strong diffuse positivity for cluster of differentiation (CD) 34, as well as expression of CD99 and vimentin. More recently, nuclear signal transducer and activator of transcription 6 (STAT6) expression has emerged as a highly sensitive and specific marker for SFT [4]. In contrast, these tumors are typically negative for S-100 protein, cytokeratin AE1/AE3, SMA, CD117, CD31, and desmin [4]. This immunophenotypic profile is essential for differentiating SFT from other spindle cell tumors, including angiomyolipoma, leiomyoma, inflammatory myofibroblastic tumor, and gastrointestinal stromal tumor [4].

Herein, we report the case of a 26-year-old man presenting with a painless, firm, and mobile scrotal mass that had progressively enlarged over a two-year period. We also discuss the clinical presentation, radiologic findings, histopathological features, and immunohistochemical profile to improve recognition and understanding of this rare entity.

## Case presentation

A 26-year-old man presented with a painless left testicular mass that had progressively increased in size over the past two years. The patient denied any history of trauma, unintentional weight loss, fatigue, back pain, sexual dysfunction, or urinary or fecal incontinence. His family history was negative for testicular or other malignancies. On admission, vital signs were within normal limits. Physical examination revealed a firm, oval-shaped mass in the left hemiscrotum, measuring approximately 60 mm (longitudinal) and arising from the paratesticular region. No associated inflammatory changes, inguinal lymphadenopathy, or gynecomastia were observed.

**Figure 1:**
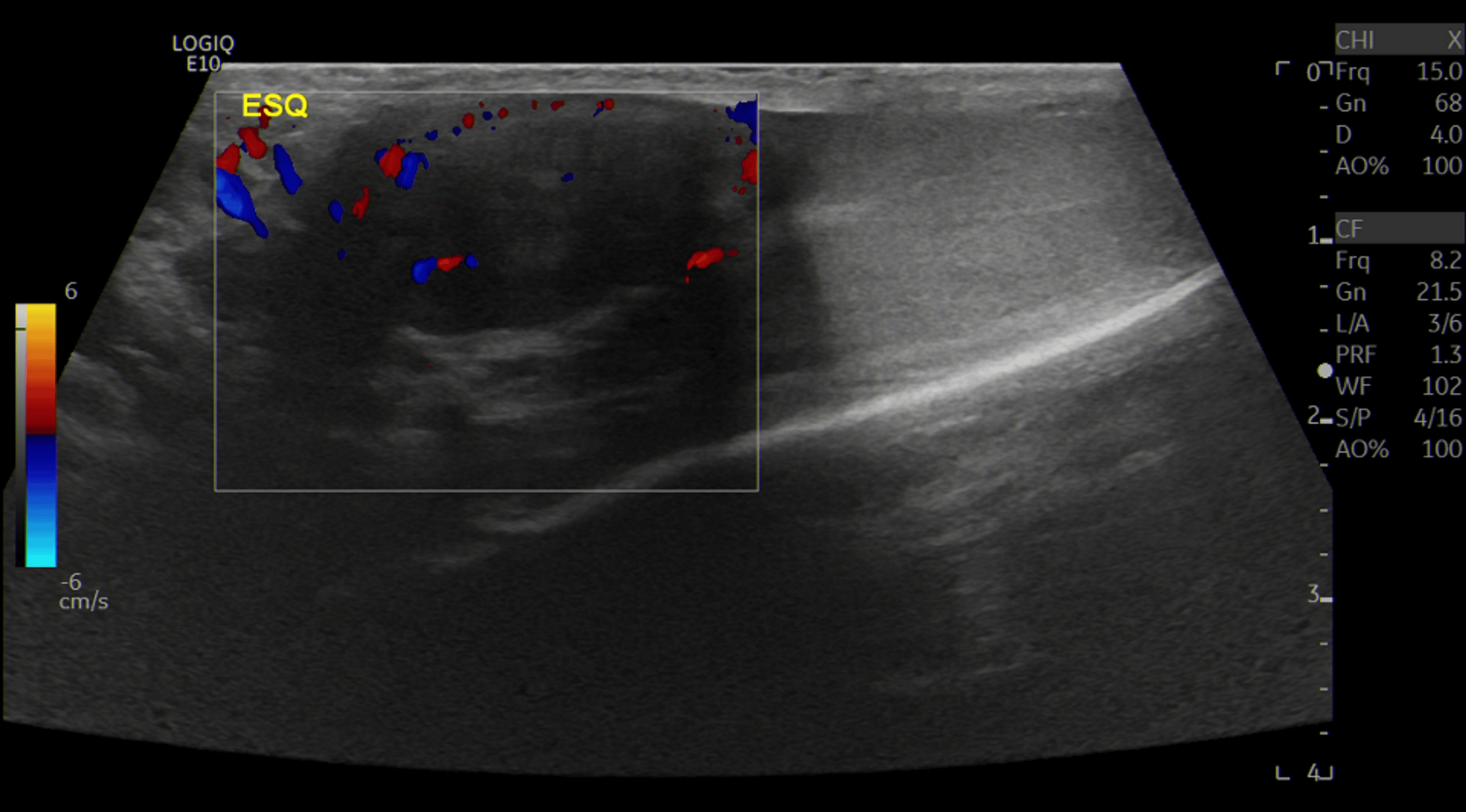
Scrotal ultrasonography showing heterogeneous and infiltrative left paratesticular mass, with positive color flow Doppler.

**Figure 2:**
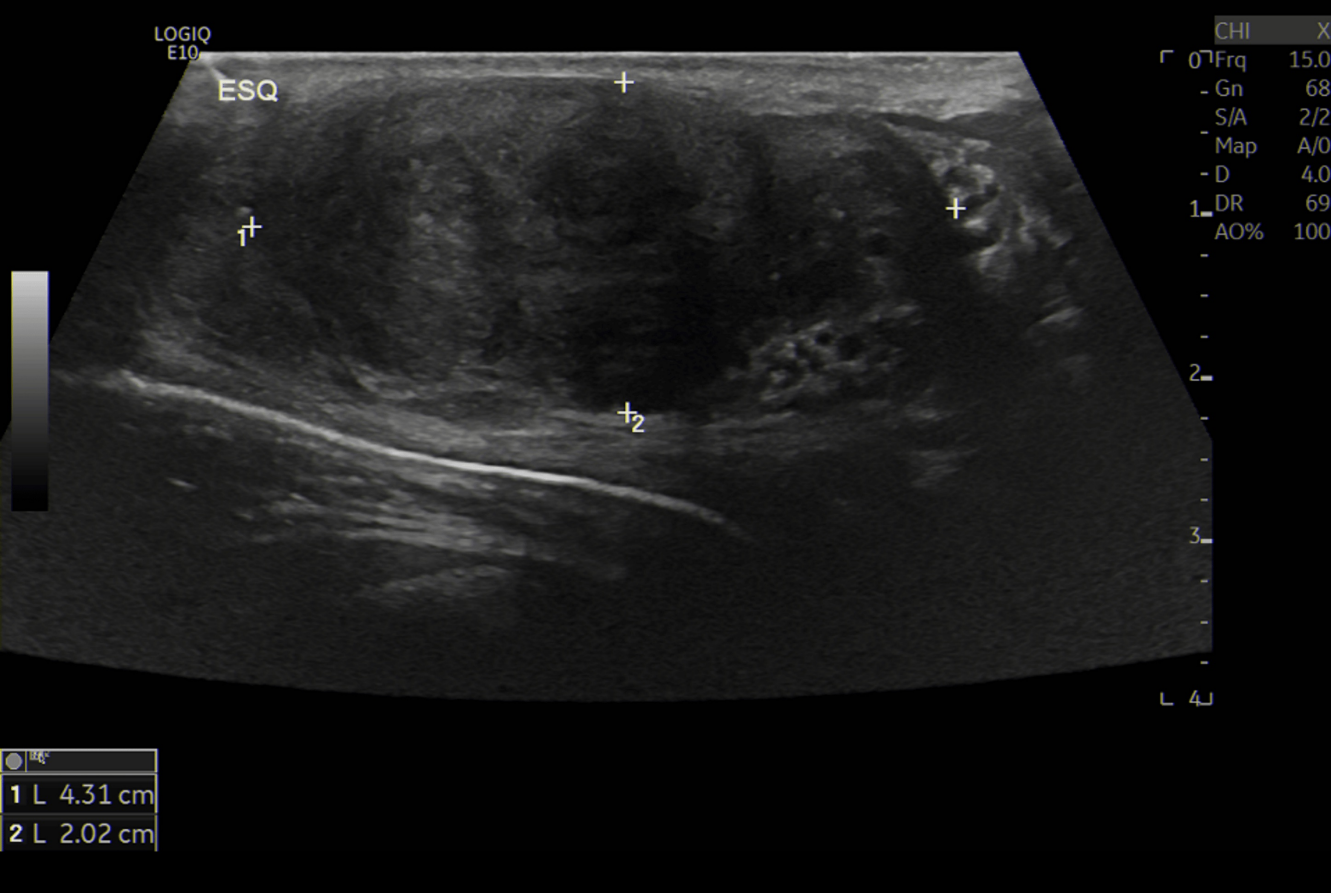
Scrotal ultrasonography-B-mode showing heterogeneous and infiltrative left paratesticular mass measuring 43 x 20 mm (longitudinal x anteroposterior)

Serum tumor markers were within normal limits, including beta-human chorionic gonadotropin (β-hCG) (< 0.2 mIU/L) and alpha-fetoprotein (AFP) (1.98 ng/mL), while lactate dehydrogenase (LDH) was elevated (698 U/L). Scrotal ultrasonography demonstrated a heterogeneous infiltrative left paratesticular mass measuring approximately 43 × 20 mm (longitudinal x anteroposterior) (Figure 1, Figure 2). Contrast-enhanced computed tomography (CECT) of the chest, abdomen, and pelvis showed no evidence of lymphadenopathy or distant metastasis.

**Figure 3:**
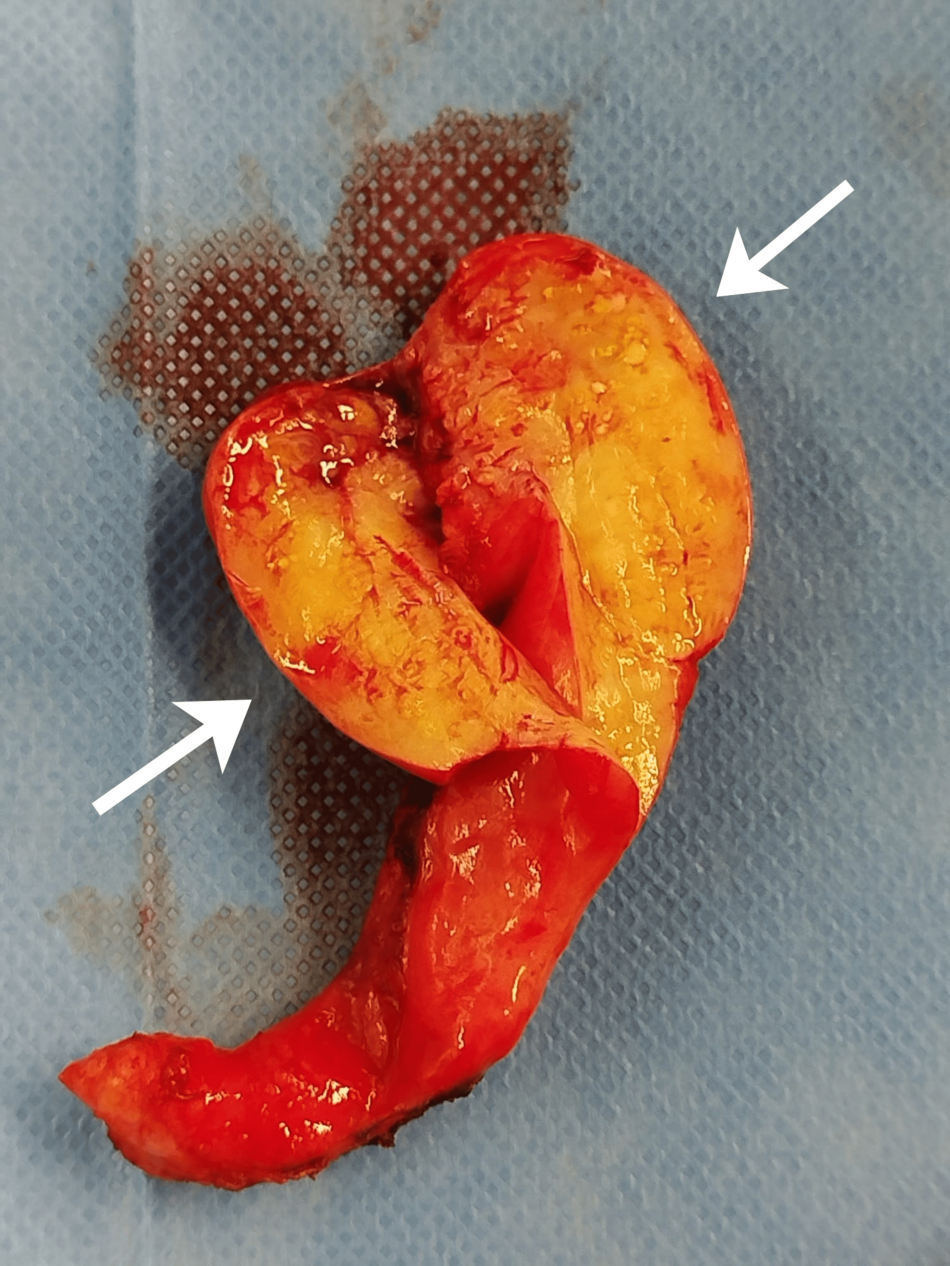
Gross specimen Well-circumscribed encapsulated nodular heterogeneous whitish mass without evidence of hemorrhage or necrosis (white arrow)

The patient subsequently underwent left radical orchiectomy without perioperative complications. Gross examination revealed a well-circumscribed, encapsulated, nodular, heterogeneous whitish mass arising from the left paratesticular tissue, measuring 103 × 21 mm (longitudinal × anteroposterior). The spermatic cord was not included in these measurements. No evidence of hemorrhage or necrosis was identified (Figure 3). Clinical and ultrasonographic measurements represent gross estimates and may be subject to significant inter-operator variability. This could account for the difference in clinical and ultrasonographic measurements with that of the gross specimen.

**Figure 4:**
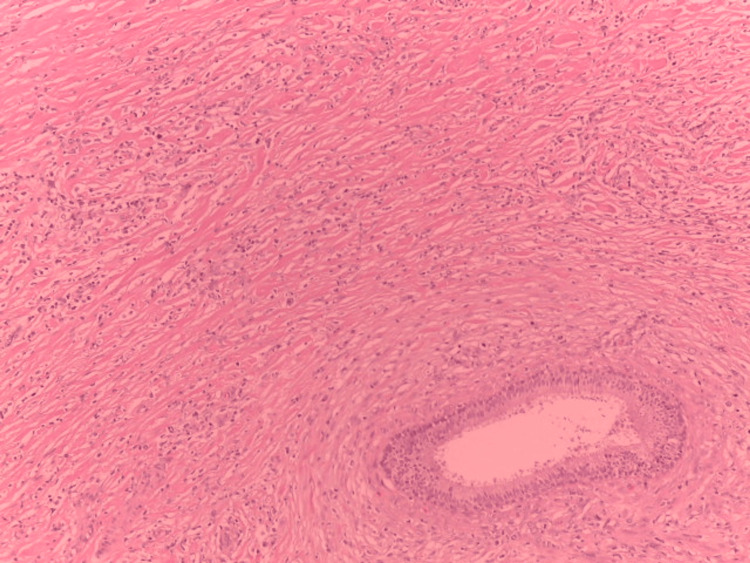
Histopathological specimen (H&E staining) showing spindle cell neoplasm with variable cellularity and a patternless architectural arrangement without cytologic atypia or necrosis. H&E: hematoxylin and eosin

**Figure 5:**
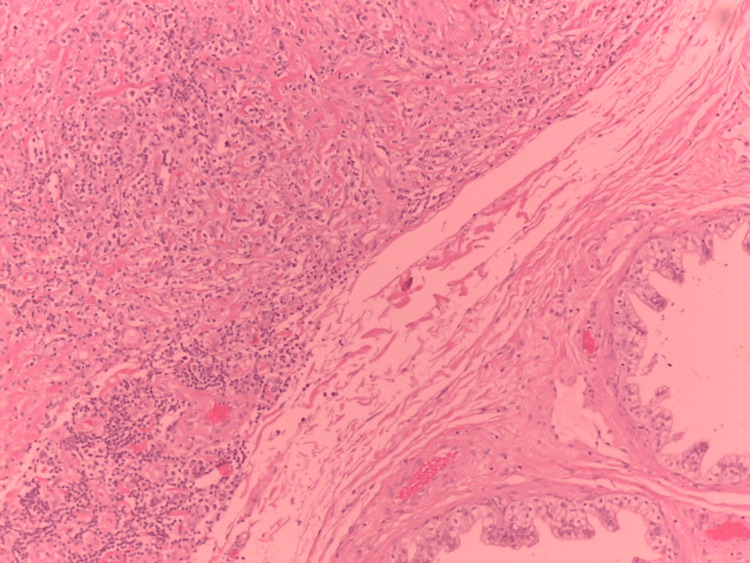
Histopathological specimen (H&E staining) showing no evidence of extratesticular extension H&E: hematoxylin and eosin

Histopathological evaluation demonstrated a spindle cell neoplasm with variable cellularity and a patternless architectural arrangement, without evidence of cytologic atypia, necrosis, or seminiferous tubule involvement (Figure 4). No extratesticular extension was identified (Figure 5).

**Figure 6:**
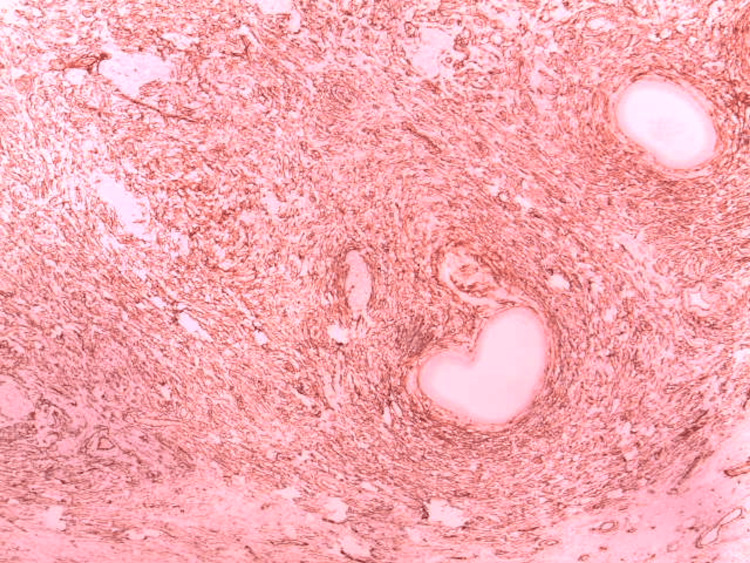
Immunohistochemistry specimen showing diffuse tumor cell positivity for CD34. CD: cluster of differentiation

Immunohistochemical analysis showed diffuse tumor cell positivity for CD34 and negativity for desmin, cytokeratin AE1/AE3, epithelial membrane antigen (EMA), carcinoembryonic antigen (CEA), human melanoma black 45 (HMB45), S-100 protein, and smooth muscle actin (SMA) (Figure 6). This immunophenotypic profile is essential for differentiating SFT from other spindle cell tumors.

These findings supported the diagnosis of SFT. The mitotic index was low (1 per 10 high-power fields), and the Ki-67 proliferation index was less than 5%. At the six- and twelve-month postoperative follow-ups, the patient remained asymptomatic, with no evidence of local recurrence or metastatic disease. Ongoing surveillance was planned.

## Discussion

SFTs are uncommon mesenchymal neoplasms of fibroblastic origin that are typically benign in nature. However, a small proportion of cases exhibit malignant behavior, with studies suggesting that approximately 10-15% may undergo malignant transformation [1]. SFTs most commonly occur in middle-aged adults and show no clear sex predilection. They classically arise from the pleura but have also been described in a wide range of extrapleural locations, including the upper respiratory tract, lungs, mediastinum, nasal cavity, paranasal sinuses, breast, meninges, liver, and pelvic cavity [2].

Genitourinary involvement is particularly rare, with the kidney and bladder being the most commonly affected sites [3]. Paratesticular localization represents an exceptionally uncommon presentation, with only a limited number of cases described in the literature. These lesions may arise from paratesticular structures such as the tunica vaginalis, epididymis, tunica albuginea, and spermatic cord. Although the exact etiology remains uncertain, some studies suggest a possible association with prior infection or trauma [5].

Clinically, paratesticular SFTs usually present as slow-growing, painless masses that gradually enlarge over time. Larger lesions may become symptomatic due to mass effect. In patients with suspected scrotal pathology, ultrasonography is typically the first-line imaging modality. However, definitive diagnosis relies on histopathological examination combined with immunohistochemical analysis [1].

Histologically, SFTs demonstrate characteristic features that are generally consistent across different anatomical sites [2]. These tumors are composed of spindle-shaped cells arranged in a so-called “patternless pattern,” with alternating hypercellular and hypocellular areas embedded within a collagenous stroma. A prominent vascular network with branching, staghorn-like vessels is frequently observed [6]. Tumor cells typically exhibit oval nuclei, inconspicuous nucleoli, and scant cytoplasm, and mitotic activity is usually low [2].

Immunohistochemistry plays a crucial role in confirming the diagnosis and distinguishing SFT from other spindle cell neoplasms [2,4]. Tumor cells commonly demonstrate strong diffuse positivity for CD34, as well as expression of CD99 and vimentin. More recently, nuclear STAT6 expression has emerged as a highly sensitive and specific marker for SFT. These tumors are typically negative for S-100 protein, cytokeratin AE1/AE3, SMA, CD117, CD31, and desmin [4].

Accordingly, SFT should be included in the differential diagnosis of spindle cell tumors arising in the paratesticular region, particularly when CD34 positivity is identified [2].

Although most SFTs are benign and well-circumscribed, complete surgical excision remains the treatment of choice. Despite their generally favorable prognosis, approximately 10-15% of extrapleural SFTs may demonstrate recurrence or metastatic behavior. The limited number of reported cases in the literature does not provide sufficient data regarding long-term follow-up of these tumors, resulting in a lack of robust evidence to guide management. Nevertheless, based on their behavior in other anatomical locations, paratesticular SFTs with low proliferative activity appear to have a low risk of local recurrence or metastatic dissemination [7].

## Conclusions

Paratesticular SFT is an exceptionally rare mesenchymal neoplasm of fibroblastic origin. Although it generally follows a benign clinical course, its rarity and its overlapping features with other spindle cell tumors of the paratesticular region make accurate diagnosis challenging. Ultrasonography is the initial imaging modality of choice in the evaluation of testicular and paratesticular masses; however, definitive diagnosis of SFT relies on histopathological examination combined with immunohistochemical analysis.

Diffuse positivity for CD34, together with the characteristic patternless architecture and a supportive immunoprofile, is central to confirming the diagnosis and distinguishing SFT from other spindle cell neoplasms of this region. Complete surgical excision remains the treatment of choice and is generally curative. Nevertheless, given the potential risk of malignant transformation and recurrence, long-term clinical follow-up is recommended. Increased awareness of this uncommon entity may contribute to its earlier recognition and to the appropriate management of paratesticular SFTs.
